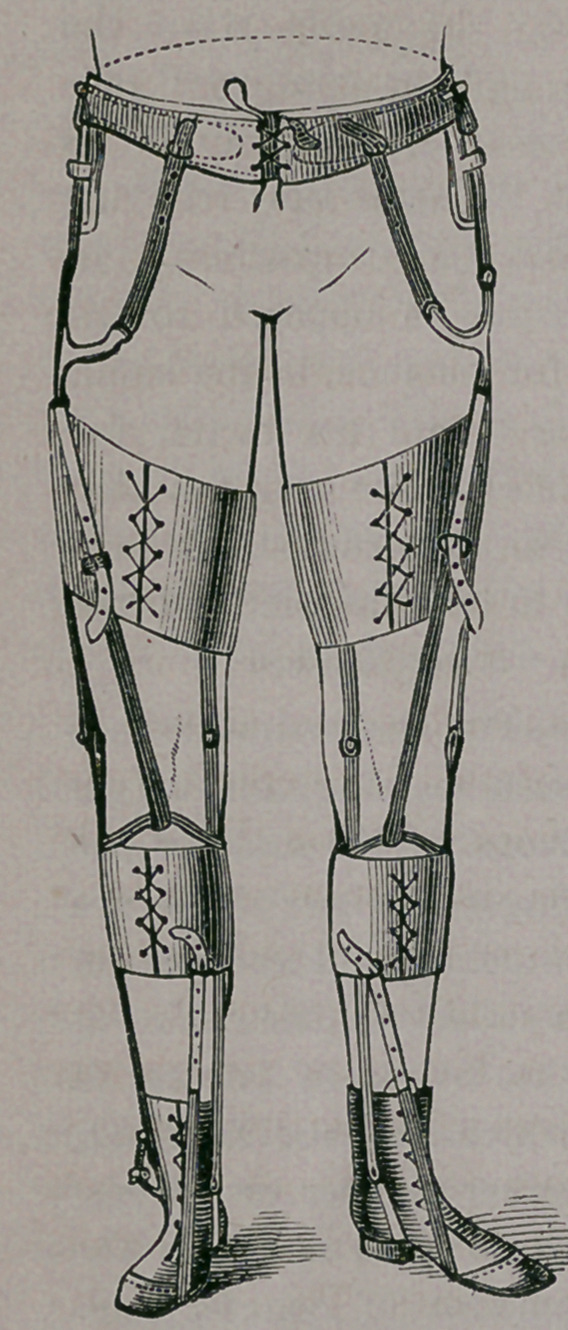# Paralysis of Children

**Published:** 1877-04

**Authors:** 


The Bistoury.

ELMIRA, N. Y., APRIL, 1877.

The BISTOURY is published Quarterly, upon the 1st of April, July, October and January, at Fifty-five Cents a year, in advance.

### PARALYSIS OF CHILDREN.

Deformities, particularly of the extremities of children, is a condition for which the orthopædic surgeon is frequently consulted. It is common to all ages—from the teething infant to the boy or girl about entering upon majority. Paralysis manifests itself in multitudinous forms—in contractions of the muscles of the hands, arms, feet, legs, neck, or upon various parts of the body. Club foot, club hand, wry-neck, contraction and consequent drawing up of the muscles of the limbs in such a manner as to produce hideous deformities. We have seen children with their limbs so contracted as to resemble more a bundle of skin and bones, bent and twisted into the smallest possible space, than that of a living person.

The cause is not always apparent. Oftentimes it can be traced to some of the disorders common to childhood — teething, scarlet fever, measles, whooping cough, and the like.

In older children, to exposure to cold, over-exertion in their sports and then sudden chilling of their persons. But from whatever cause, the consequences are of the most distressing character both to the parents and child.

Some of the deformities arising from paralysis are curable and some are not. How to distinguish the one form from the other can only be determined by the surgeon, so that we will not attempt to give any instruction upon this point. We desire simply to call the attention of parents to the fact that, deformities from contracted limbs, crooked hands and feet, are curable, so that they may not allow their maimed little ones to continue in their helplessness not knowing that a cure is at hand.

Often, the contraction of the muscles is so great as to cause great pain—the finger nails being thrust into the palms of the hand, the arms severely bent upon themselves, or the knees being drawn up to the chin. Of course, lighter degrees of the malidy exist, such as a contraction of the fingers or toes, or the flexion of the fore arm, or the turning of the neck.

Instant relief is given to the more severe forms by use of the surgeon's knife. A very delicate blade is thrust under the skin, making an inscision so small as to be scarcely observable, while the pain is almost unnoticed, and the contracted tendon is severed, at once restoring the limb to its former usefulness and at the same time relieving all pain that resulted from the contraction. In milder cases, a counter force is opposed to the contracting muscles. As for instance, in the hand, where the tendons closing it are contracted for want of power, or paralysis of the muscles that open the hand, a glove is so constructed, with artificial rubber muscles, as to take the place of the paralyzed ones, as seen in our illustration:

The elastic muscles are seen passing through loops to the ends of the fingers, where they are attached, and terminate in a series of little chains at the wrist, by means of which they are fastened, either shorter or longer, (depending upon the amount of counter-contraction desired) to a band about the wrist. In this manner, the disposition to spasm of the contracting muscles is prevented, while the enfeebled muscles of the back of the hand are exercised and strengthened.

Should the paralysis occur among any muscles of the body, causing a crooked spine or wry-neck, either the muscles can be severed that are causing the deformity, as before described, or a suitable appliance adjusted to overcome the action of the contracting muscles, while the enfeebled ones are assisted.

In children suffering from paralysis, it is very common to see the limbs horridly distorted and cramped, so that locomotion becomes wholly impossible. By the application of the delicate instrument which the surgeon uses for this purpose, the contracting tendons are quickly severed—cut without a particle of pain or the appearance of a drop of blood. No gaping wound is left to annoy and irritate the child while it is healing. All is accomplished and hidden under the skin, so that no mark, save a delicate puncture in the skin, is left to indicate the surgeon's visitation, the deformity as speedily disappears and the child is placed in an apparatus like the one here figured, when walking becomes at once possible.

Should the case be a mild one, often the adjustment of the apparatus alone, is sufficient to accomplish the cure, fitted as it is with elastic muscles like the appliance first described. Running from the shoes up the inside and outside of the leg, is a light steel brace, with a joint at the ankle, knee and hip. About the waist is a stout, steel band, suitably padded, to which these rods are rivited. About the leg and thigh are broad, padded bands to give further support, and from these are seen running the elastic muscles to give the necessary extension. One is seen passing from the leg to the toe, to raise the foot—another from below the knee to the hip, to throw the leg forward, &c.

We introduce these figures simply to show to what perfection orthopædic appliances are brought at this day, to overcome all manner of deformities, so that the general reader may familiarize himself with them and perhaps construct a simple appliance to overcome some slight deformity in a member of his own family. At all events, it is best to know that such ailments are remediable, and that any skillful orthopædic surgeon is at all times prepared to furnish the means necessary to accomplish the cure.

## Figures and Tables

**Figure f1:**
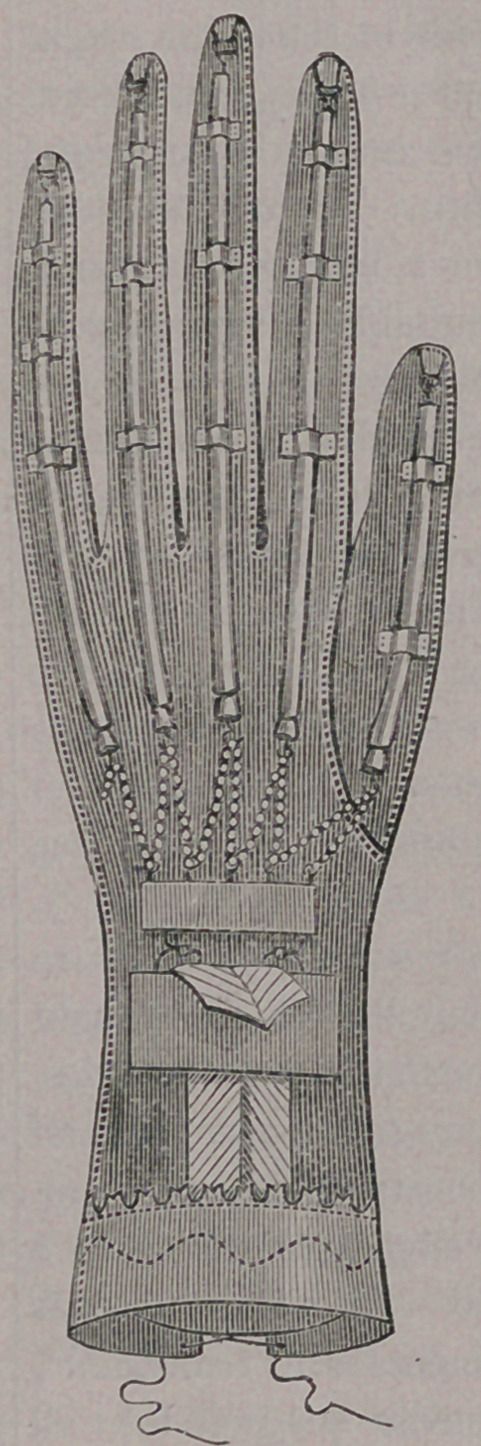


**Figure f2:**